# Future Implications of Using Registered Dietitians in Multidisciplinary Polycystic Ovary Syndrome Treatment

**DOI:** 10.3390/healthcare6040144

**Published:** 2018-12-08

**Authors:** Wendy M. Wolf, Rachel A. Wattick, Pamela J. Murray, Melanie Clemmer, Melissa D. Olfert

**Affiliations:** 1 Apex Family Medicine, Denver, CO 80209, USA; wendywolf02@gmail.com; 2 Department of Human Nutrition and Foods, West Virginia University, Morgantown, WV 26506, USA; rawattick@mix.wvu.edu; 3 Department of Pediatrics, Section of Adolescent Medicine, School of Medicine, West Virginia University, Morgantown, WV 26506-9214, USA; pmurray@hsc.wvu.edu; 4 Department of Obstetrics and Gynecology, School of Medicine, West Virginia University, PO Box 9186 Morgantown, WV 26506-9186, USA; mclemmer@hsc.wvu.edu

**Keywords:** multidisciplinary treatment, nutrition interventions, treatment barriers, PCOS, nutrition education

## Abstract

Polycystic ovary syndrome (PCOS) is the most common reproductive endocrine disorder in females with insulin resistance playing a key role in pathogenesis. The objective of this study was to investigate current trends and future implications of multidisciplinary PCOS clinics with inclusion of dietitians. A two-phase, formative investigation on practitioners was conducted through an anonymous survey followed by focus groups. Survey respondents included 261 health care providers from around the world; the majority (59%) representing multidisciplinary teams. Focus group participants included four dietitians, three physicians, a health psychologist and a licensed nutritionist. Primary barriers for future multidisciplinary clinics included: money/resources, insurance reimbursement, and difference of opinions. Potential advantages included: more comprehensive and integrated care, greater convenience/efficiency, and better long-term outcomes. A majority of respondents (89%) stated that dietitians should be ‘involved’ or ‘highly involved’ in treatment. The greatest challenges for dietitians include insurance, limited disease knowledge, and lack of referrals. Most providers agreed that multidisciplinary clinics would lead to a better prognosis. A greater emphasis needs to be placed on educating professionals on the importance of nutrition counseling. Access to educated dietitians is likely the best way to ensure that PCOS patients have access to lifestyle interventions.

## 1. Backgrounds

Polycystic ovary syndrome (PCOS) is thought to be the most common endocrine disorder found in women [1,2]; PCOS is most often characterized by an imbalance of the sex hormones [1], impacting women of all races and ethnicities who are of reproductive age [2]. Common symptoms include irregular menstrual cycle, ultrasound abnormalities of increased ovarian volume and follicle count, and hirsutism (male-patterned hair growth) [2]. Furthermore, a statistical report by Futterweit estimated that 50 to 75% of women with PCOS are unaware that they even have this syndrome [3]. Features of the syndrome may also include infertility, insulin-resistance, impaired glucose tolerance (Type 2 Diabetes), dyslipidemia, and cardiovascular disease due to increased risk factors [1,4]. Additionally, one of the originally described comorbidities of PCOS was obesity, however obesity or overweight is not obligatory in PCOS; thus, women with a lean figure and android fat distribution are usually termed lean PCOS women. The etiology of PCOS is not completely understood and there is no known cause, although a genetic component and lifestyle influences have been identified [1,2,4]. Due to the heterogeneous and multifactorial nature of PCOS symptoms there is a lack of a clear universal consensus regarding the definition and diagnostic criteria [1,5]. General estimates of the prevalence of PCOS range from 3–10% [6,7].

With the strong association between obesity and insulin resistance, weight loss is supported by the Androgen Excess Polycystic Ovary Syndrome Society as part of lifestyle intervention as the first-line treatment for overweight and obese women with PCOS [8]. Studies have shown that even a modest amount of weight loss, 5 to 10% of body weight, can reduce the severity of the symptoms for PCOS [8]. Current research supports that achieving weight loss or preventing weight gain is best done with assistance from a multidisciplinary team that includes dietary modifications, exercise, and behavioral therapy [9]. Geier et al. [10] has shown that the patients who had the most success with weight loss had met with both the dietitian and health psychologist at an adolescent (average age at first visit 15.9 years) multidisciplinary PCOS clinic [9,10].

There is limited literature about multidisciplinary PCOS clinics and the efficacy of their treatment. The limited research documenting the outcomes of multidisciplinary PCOS clinics has demonstrated increased weight loss, high patient satisfaction rates, and high retention rates [10,11].

PCOS treatment typically involves medication and lifestyle interventions to best manage the symptoms and disease risks associated with PCOS. Lifestyle interventions include a combination of dietary changes, increased physical activity, stress management and smoking cessation. Current literature supports the use of lifestyle intervention as the first-line treatment for patients with PCOS, especially those who are overweight and obese [12].

The current literature lacks the perspectives of health care providers on PCOS and evidence of the benefits of dietitians in PCOS treatment. The objective of this study was to investigate the current trends and future implications of multidisciplinary PCOS clinics, emphasizing the importance and challenges for dietitians.

## 2. Materials and Methods

### 2.1. Study Design

This was a two-phase formative study. The first phase was a preliminary cross-sectional, anonymous, Internet survey (Qualtrics, Provo, Utah) that approached a broad category of health care providers to assess current trends in PCOS treatment and explore implications for future multidisciplinary clinics though qualitative and quantitative data. The second phase consisted of a series of focus groups designed to obtain qualitative data that was focused on the utilization, importance and challenges of involving dietitians in the treatment of PCOS. The Institutional Review Board of West Virginia University approved the study protocol.

### 2.2. Participants

Phase one contacted health care providers who currently provide care to PCOS patients were recruited for the survey using four list serves: Society for Adolescent Health and Medicine (Oakbrook Terrace, IL, USA), North American Society for Pediatric and Adolescent Gynecology (Mt. Royal, NJ, USA), Society of Assisted Reproductive Technology (Birmingham, AL, USA)—American Society of Reproductive Medicine (Birmingham, AL, USA), and EmbryoMail, various LinkedIn Groups, individuals identified by their research in the field or their involvement with existing PCOS treatment centers, and participant referrals. A total of 261 health care providers initiated the survey with a 47% completion rate.

Phase two was a descriptive study that relied on a purposive, non-probability sample that was selected based upon theoretical sampling [13]. An invitation was sent out to responders from the original survey who submitted their contact information and resided in the United States (*n* = 22) inviting them to participate in a focus group. Those respondents were encouraged to refer additional health care providers leading to an additional 16 providers contacted. A total of nine providers engaged in the series of focus groups providing a participation rate of 24% of those contacted.

The sample size for neither phase was pre-determined and recruitment persisted throughout the duration of the data collection. All survey participants implied consent and completed the survey voluntarily and all focus group participants gave oral consent and received a $25 gift card after the conference call. West Virginia University Institutional Review Board approved the study prior to data collection and analysis.

### 2.3. Survey Instrument

The Internet-based survey consisted of 30 multiple-choice, multiple-response, and open-ended questions targeting information on their demographics, current treatment facility and approach, and perspectives about future multidisciplinary clinics. This survey was designed based on current literature reviews and existing multidisciplinary clinic data. Professionals in the field including a physician, fertility specialist, dietitians, and master’s students reviewed the survey for feedback. The final survey was released and left open for two months (May and July 2013).

### 2.4. Focus Group Methodology

The primary researcher moderated each focus group and three note takers were kept consistent throughout the series. All of the researchers involved completed human subjects’ research Collaborative Institutional Training Initiative training. Similar providers were placed together to promote group cohesiveness [14,15] and compatibility [16,17]; for example, physicians were paired with other physicians and dietitians were kept together as much as possible. The moderator followed a question guide developed by the research team based on the findings of the surveys and the current literature. During the focus group, participants were asked to respond to a series of open-ended questions (Table 1).

During the study, participants engaged with others via teleconference. All focus groups were audio-recorded for the primary researcher to transcribe them verbatim.

### 2.5. Data Analyses

Frequencies and measures of central tendencies from the survey were analyzed using SAS software (SAS 9.3, SAS Institute, Cary, NC, USA).

The focus group verbatim transcript was compared with the note-takers’ notes to examine for any discrepancies. The final transcription was analyzed to identify themes and sub-themes and how extensive the participants discussed topics. The transcriptions were reduced to exclude any unnecessary words to facilitate the identification of themes efficiently. Braun and Clarke’s method [18] for thematic analysis was used to sort through the reduced data. By using thematic analysis we are relying on the content analysis, which focuses on intentionality and implications of the context [18]. After the themes were identified and coded they were sorted and paired accordingly. Themes are identified with re-occurring context noted and theoretical saturation was reached when new analysis only produced codes that fit into existing categories. Glasser defined the theoretical saturation as met once the properties and dimensions of the categories were fully explained and new data fit into existing themes [13].

## 3. Results

### 3.1. Survey Results

There was a total sample size of 261 health care providers who provided care to individuals with PCOS from a variety of specialties representing various settings of care (Table 2). The sample was 78% female and 22% male.

### 3.2. Current Clinic Descriptions

Fifty-nine percent (*n* = 79) of the responders treated PCOS in a multidisciplinary setting, defined as utilizing at least two health care providers from different specialties, whereas (*n* = 56) 41% were independent providers. For those responders who were part of a multidisciplinary team the breakdown of other specialties involved are listed in Table 3.

### 3.3. Existing Clinic Outcomes

Responders (*n* = 88) were asked to list the top one or two items that their facility could improve upon. The most common theme identified, at 34%, was to incorporate more multidisciplinary involvement with more integration and/or communication. The second most popular theme, with 30% was to expand nutrition and/or exercise programs to support weight loss. Improving or eliminating access barriers that prevent treatment of patients (10%) was also a common theme. The three most common access barriers were identified to be patient waiting time, cost and health insurance.

Responders (*n* = 87) were also asked to list the top one or two items that their facility does well. 21% of responders stated the top were the treatment/management of symptoms, 21% said nutrition/lifestyle changes, and 20% of responders stated patient education/counseling. With 17%, multidisciplinary collaboration with other providers was the fourth most common theme.

### 3.4. Future Implications for Multidisciplinary Clinics

The most common potential barrier to future multidisciplinary clinics noted by survey responders were money/resources followed by the lack of insurance reimbursement. The greatest potential advantage noted was the increased ability to provide comprehensive and integrated care to address all aspects of PCOS. See Table 4 for additional responses.

In order to determine the perceived benefits and importance of the involvement of specialties in future multidisciplinary clinics, responders were asked to rate the importance of involvement of provider types (Table 5). Dietitians received the highest perceived value of responders who felt they should be ‘highly involved’, followed by physicians.

### 3.5. Focus Group Results

The focus group participants included health care providers that fit in to one of the three following categories: registered dietitians, physicians, or other practitioners. All providers treated patients with PCOS on a regular basis and had between 7–25 years of experience. We conducted a series of focus groups via teleconferencing with a total of nine participants; two were male and seven were females. We spoke with three physicians, two pediatric endocrinologists and one internal medicine/adolescent medicine physician, four registered dietitians, one health psychologist, and one licensed nutritionist/certified nutrition specialist. These providers primarily worked in large metropolitan areas spread across the United States. The majority (*n* = 6) of participants worked in multidisciplinary facilities where they shared a location with other types of providers; whereas the remaining three providers were solo providers who were in their own practice facility.

Overall, these providers felt that dietitians are highly overlooked in the treatment of PCOS. The most common barriers for dietitians included lack of insurance, lack of PCOS-specific knowledge and the lack of physician referrals. Key themes are described in Table 6.

## 4. Discussion

The current formative study investigated the opinions of health care providers who frequently treat PCOS about potential implications for the role of dietitians in the multidisciplinary treatment of PCOS. Potential benefits of specialized individualized, and multidisciplinary care were explored. There were a variety of challenges preventing dietitians from being involved to the fullest capacity with the treatment of PCOS.

Our survey found that 71% of individuals involved with a multidisciplinary clinic involved a dietitian, but a study on United Kingdom dietitians who treated PCOS found that only 36% worked jointly with other health professionals [19]. Because our study advertised assessing multidisciplinary PCOS, it is likely that our sample attracted a higher percentage of multidisciplinary providers than is truly representative. Our results suggested lower promotion of lifestyle interventions from physicians than a prior study assessing clinical variability in approaches to PCOS via a similar Internet survey that was distributed to the North American Society for Pediatric and Adolescent Gynecology members, which found that 90% of physicians recommended diet modification/exercise for a first-line treatment [20]. While our study included the North American Society for Pediatric and Adolescent Gynecology list serve, we also included other outlets for recruitment, which resulted in a different demographic representation inclusive of endocrinologists on top of more typical gynecologists and adolescent medicine physicians. Discrepancies between the studies may be due to the fact that physicians surveyed by Bonny et al. did not actually refer patients to see a dietitian, but rather just recommended nutrition-related modifications. Although this is speculation, it is supported by the other studies that saw very minimal interactions (as low as 17%) with the dietitians in comparison with the high rates that claimed nutrition recommendations (90%) in the Bonny study [21,22].

The limited accessibility to dietitians was addressed in our focus groups and responses showed that patients were less likely to see a dietitian if they were located in facility separate from their physician or seeing the dietitian required a separate visit. Our focus group results overall suggested similar findings to what current statistics in studies done on multidisciplinary clinics show in terms of patients seeing health psychologists and dietitians on top of a gynecologist and/or endocrinologist, but these findings still reflect a much higher percentage of patients seeing a dietitian than those not treated in multidisciplinary clinics [21,22].

Results of our study supported evidence that PCOS is a complex and heterogeneous disorder that requires multidisciplinary treatment including both lifestyle, diet, and behavior modifications to manage patients in the ideal way [23], but responses in our focus groups signified that access to nutritional intervention counseling is very limited for the majority of PCOS patients. Some research shows that only 15% of patients with PCOS had ever seen a dietitian, and that number was further reduced to 3% for patients who had had more than two appointments with a dietitian. When assessing the differences in accessibility to dietitians and nutritional interventions in overweight and obese women compared to women with lean PCOS, the focus group results found that lean PCOS is often overlooked and the obese PCOS cases are typically more symptomatic, making them more obvious referrals despite the perception of dietary management being of equal importance in both groups. This seems to be a common disparity, as results from Jeanes et al. found that overweight women with PCOS were more likely to receive advice from a dietitian (21%) than lean PCOS women (10%); similar results were seen in the percentage of women with PCOS receiving dietary advice from a physician, with 25% and 17% respectively.

Common barriers that prevented some of the patients in a multidisciplinary clinic setting from seeing the dietitian and health psychologist seem to be the denial of access by referring Health Management Organizations or insurance providers followed by the patient refusing the visit due to perceived stigma or simply the fact that they did not want to consider dietary interventions. Another barrier that was reported by Geier that was not mentioned in our study was that some patients had a prior therapeutic relationship with a psychologist or psychiatrist that was not affiliated with this multidisciplinary clinic. It was noted in this study that there was a lack of perceived benefit from patients with PCOS that had a normal body mass index (BMI), even though a few still had insulin resistance [10]. This was similar to the concept addressed in the focus group that they already know what the dietitian is going to tell them or that they think because they are already lean, diet changes will not help them. The study by Geier et al. was a retrospective study that had no consistent documentation for refusal reasons.

Our study noted that a major challenge for dietitians in the United States treating PCOS was the lack of focused PCOS education for dietitians. Comparatively, data collected amongst United Kingdom dietitians who treated PCOS showed only 34% reported feeling well informed of the PCOS literature, and 64% believed that there was insufficient evidence regarding the dietary management of PCOS in 2009, support this finding [20]. Additionally, our study found that many dietitians do not even receive referrals from physicians for patients with PCOS. Potential reasons for the lack in referrals include the lack of confidence that physicians have in the success of lifestyle intervention methods. Some data does show that physicians do not believe obese patients will actually lose a significant amount of weight, and that very few of these physicians believe they are usually successful in assisting obese patients lose weight. When assessing the importance of dietitians our focus groups conveyed that physicians should not be the ones fully responsible for dietary interventions because they lack the training and the time it takes to facilitate the change. A significant amount of patients in a Humphreys et al. study claimed receiving their nutrition information only from the Internet or their endocrinologist whom they only saw twice a year [22].

Lifestyle intervention counseling is felt to be important, but is infrequently incorporated in a systematic way within the treatment of PCOS. There are many challenges to successfully incorporating dietitians but with improvements in education and insurance they can play an integral role in PCOS. Our study and the findings of others found that despite the fact that weight loss and weight maintenance are vital to reducing of symptoms and long-term risk for PCOS, the general consensus is that the education and support given to these patients is inadequate.

To our knowledge this is the first study that seeks to gain insight from a mix of health care providers who frequently treat PCOS patients on the potential of multidisciplinary clinics and the challenges of involving dietitians in the care of PCOS.

### Strengths and Limitations

There are some limitations to this study. The sample used in this study was relatively small. Our survey sample did not allow for an associative analysis, to explore the potential association between different providers or type of treatment. The survey relied on self-reported data with no means of verification of credentials or experience. Several limitations were imposed on this study that accompany the nature of convenience sampling and focus groups. Generally, anonymous internet questionnaires may not be most effective way to capture perceptions. This study did seek the opinions of experts and it was not meant to be generalizable to the entire health care provider population. It was not the intent of this study to generalize the findings to the entire population of health care providers, but to gain feedback from the leaders in the field of PCOS care on the impact and barriers regarding nutritional interventions and multidisciplinary PCOS treatment. It is possible that different experts would have different opinions with regard to PCOS but after reaching saturation it is unlikely the results would have been significantly affected. This purposive sample provided access to rich qualitative data that cannot be gathered though traditional surveys.

## 5. Conclusions

Polycystic ovary syndrome is a complex condition that requires the expertise of multiple provider types to treat the syndrome in its entirety. Most providers agreed that multidisciplinary clinics would ultimately lead to a better prognosis for PCOS patients. The perceived barriers that prevent clinics from becoming multidisciplinary would need to be well defined, but providers indicate enthusiasm for the opportunity to implement a multidisciplinary approach. A greater emphasis needs to be placed on educating the medical community, including dietitians and physicians, on the importance of specialized nutrition counseling and lobbying for insurance reimbursement. Having access to dietitians educated about PCOS is likely the best way to ensure that PCOS patients have access to lifestyle interventions, which is considered to be the first-line treatment for PCOS.

## Figures and Tables

**Table 1:** Outline of questions for focus group used in a qualitative study on involving registered dietitians in multidisciplinary polycystic ovary syndrome (PCOS) treatment.

Focus Group Question Outline
1. Describe any nutritional interventions that you provide to your patients?
2. How is the dietary intervention and patient care communicated between providers?
3. When is dietary intervention warranted for a patient with Polycystic Ovary Syndrome?
4. How accessible are nutritional interventions for the majority of Polycystic Ovary Syndrome patients?
5. What are some of the challenges for getting dietitians more involved with Polycystic Ovary Syndrome?
6. Do you feel like providers know and understand the value of nutritional interventions for Polycystic Ovary Syndrome patients?
7. In your career, have you seen any shift in the awareness or interest of Polycystic Ovary Syndrome?

**Table 2:** Professional characteristics of the health care providers from survey collecting data on multidisciplinary PCOS treatment clinics.

Demographics	% Selected (*n*)
**Specialty**	
Physician	66% (138)
Dietitian/Nutritionist	22% (46)
Fertility Specialist	5% (11)
Researcher	4% (8)
Mid-level Providers Nurse Practitioner, Physician Assistant)	3% (7)
Educator/Counselor	3% (6)
Other	9% (19)
**Setting for Care**	
Hospital or Clinic	66% (135)
Private Office	45% (92)
Research Facility	8% (17)
Other	4% (9)
**Population Setting**	
Urban	70% (98)
Suburban	23% (33)
Rural	6% (9)
**Location**	
United States	64% (117)
Outside of the United States	36% (67)

**Table 3:** Types of specialty providers involved in multidisciplinary PCOS clinics (*n* = 132).

Specialty	% Involved (*n*)
Dietitian/Nutritionist	71% (94)
Physician	67% (89)
Nurse	48% (63)
Fertility Specialist	35% (46)
Mid-Level Providers (NP, PA)	28% (37)
Social Worker	28% (37)
Psychologist	26% (34)
Researcher	23% (30)
Educator/Counselor	15% (20)
Physical Therapist	11% (14)
Other	17% (25)

**Table 4:** The potential advantages and barriers of future multidisciplinary PCOS clinics.

	Future Implications	Percentage (*n*)
Potential Advantages (*n* = 82)	More comprehensive and integrated care	32% (26)
	Better results/long-term care outcomes	18% (12)
	Greater convenience/efficiency	15% (12)
	Better communication between providers	15% (12)
	Increased access to more disciplines	10% (8)
Potential Barriers (*n* = 76)	Money and resources	30% (23)
	Insurance/reimbursement	26% (20)
	Difference of opinions	17% (12)
	Time (length of visit)	12% (9)

**Table 5:** Ideal involvement of specialties in future multidisciplinary PCOS clinics (*n* = 113) as perceived by health care providers.

Specialty	*n*	Highly Involved	Involved	Neutral	Occasionally Involved	Never Involved
Endocrinologist	109	48	36	6	7	3
Gynecologist	110	45	43	5	5	2
Physician (Other)	95	20	42	21	13	4
Dietitian/Nutritionist	110	59	30	6	3	2
Psychologist	105	11	45	21	15	8
Mid-Level Providers (Nurse Practitioner, Physician Assistant)	90	17	31	29	11	11
Nurse	96	19	32	30	15	4
Exercise Physiologist	95	18	40	18	9	15
Fertility Specialist	97	30	33	14	13	9
Social Worker	93	10	25	31	22	13
Physical Therapist	90	6	24	30	20	20

**Table 6:** Key themes identified by health care providers participating in focus group data collection (*n* = 9) assessing the role and challenges for involving dietitians in multidisciplinary treatment of PCOS.

Main Theme/Question	Emerging Theme with Supporting Excepts and Summary
Nutritional Interventions Provided	**By Physicians:** • Collected basic diet history, food frequency questionnaires • Providing brief nutrition education • Referred majority of patients to dietitian • *“I am not your typical physician because I do focus heavily on nutrition because it is very important in primary care”* **By Dietitians:** • Followed standard nutrition care process • Provide the bulk of the nutrition education for patients • Provide very individualized treatment depending on patients symptoms and goals • Explain and clarify information about PCOS • *“Most of these women feel like they are not being listened to by the medical community—specifically a lot their doctors just tell them to ‘eat less, exercise more’ but (are) not explaining to them.”* **By Health Psychologists:** • Explores emotion eating, food-coping mechanisms, disordered eating • Supports the dietitian and physicians in treatment • *“The dietitian providers the education, the psychologist gets the change.”*
Patient Care Communication Between Providers	**Varies by Practice Setting:** • Solo Practitioners: ⚬ Limited opportunity because treatment is ‘piece-mealed out’ ◾ No face to face communication ◾ Only email and progress notes ◾ Confidentiality barriers ⚬ *“Communication is important in any case but I think it really helps support the patients so they know that we are all on the same page and the doctors can reinforce behaviors to improve compliance and provide extra support to the patient.”* • Multidisciplinary settings: ⚬ More verbal communication ⚬ More integration of care ⚬ Little formal case management ⚬ *“I would love to have even more regular, communication!”*
Importance of Dietary Intervention for PCOS	**When Dietary Intervention is Warranted:** • All providers stated it was always important to provide nutrition intervention • Equally important regardless of Body Mass Index ⚬ *“Overweight/obese* PCOS *patients tend to be more symptomatic making them more obvious referrals”* ⚬ Lean PCOS is more often overlooked • Patients should meet with dietitian as soon as possible after diagnosis **Why Dietary Intervention is Warranted:** • It is the first-line of treatment for PCOS • To minimize long-term risk factors, insulin resistance, and symptoms **What is Actually Happening Across the United States:** • Not many women with PCOS are getting lifestyle interventions • *“Women with lean PCOS are highly overlooked by the medical community because they don’t think nutrition can help them because they are already thin even though a low glycemic index diet has been shown to increase ovulation and manage insulin abnormalities. I have seen plenty of lean PCOS women and dietary intervention absolutely helps!”*
Accessibility of Nutritional Interventions for Majority of PCOS Patients	**Not Very Accessible:** • All providers unanimously agreed • Factors that determined accessibility included: ⚬ Patients geographical location (proximity to a dietitian who understands PCOS) ◾ *“Dietitians in general are more accessible but there are just not a lot of dietitians who are well versed and experienced withPCOS.”* ⚬ Willingness of physicians to refer out ⚬ Insurance coverage/financial situation
Challenges for Getting Dietitians More Involved in the Treatment of PCOS	**Insurance** • Most common barrier identified • Varied by state • *“We are not able to effectively use dietitian because of the lack of insurance coverage.”* **Lack of PCOS Education** • Not covered in there training ⚬ *“Majority of dietitian know very little about* PCOS*, let al.one how to treat it.”* ⚬ *“There is no certification for PCOSso there is nothing across the board that provides a certain protocol for treatment.”* ⚬ *“The limited training for Registered Dietitianss on PCOS is a huge problem and the profession needs to find a way to embrace this issue”* • Very frustrating for patients if they see a dietitian who does not understand PCOS **Lack of Physician Referrals** • *“Physicians are the gatekeepers”* • *“It does not occur to most patients to seek out a dietitian to visit on their own.”* • Potential reasons for physicians not referring: ⚬ Lack of insurance coverage ⚬ Limited access to knowledgeable dietitian in the area ⚬ Not educated on the value ⚬ Quick to write off as uninterested or noncompliant ◾ *“I just had a patient tell me that a doctor was just throwing people on medication instead of also referring to a dietitian because he believes they won’t follow through, so he doesn’t even try.”* ⚬ Little confidence that lifestyle intervention will make a difference ⚬ Value pharmacological treatment, higher compliancy ⚬ Feel they have treatment covered **Lack of Follow-Through from Patients** • Lack of insurance coverage • Not ready for change • Practicality • Stigma or punishment • Overwhelmed • Already know what the dietitian is going to tell them ⚬ *“It is just food—you are just going to tell me to eat asparagus rather than a snickers bar and I already know that.”*
Importance of Involving Dietitians	**The Only Way Patients Have Access to Adequate Lifestyle Interventions** • Physicians should not be fully responsible: • Little to no training • “They can only be the experts on so many things” • Takes time • “It is so much more than handing them a 1200 kcal diet plan and telling them to exercise and lose weight; it is about trying to un-root deeply seeded behaviors that are tied to emotions.”
Understand of the Value of Nutritional Interventions	**Not as Well as They Should** • Few physicians that understand the value of nutritional interventions for PCOS but the majority do not • Many physicians overlook the increased risk for co-morbidities • *“PCOS is the most common endocrine disorder among reproductive age women and dietitians don’t even know what it is. That is a big issue!”* • *“Nutrition professionals need to be a lot better at what we do in terms of understanding PCOS and letting other providers know that we need to be involved.”* • *“Often times, withPCOS, nutrition counseling is treated like dermatology and it needs to be treated more like psychology.”*
Shift in Awareness of PCOS	**Over the Past Decade:** • More information in the lay press ⚬ Patient-driven diagnosis ⚬ More support groups and websites for patients • More awareness in medical community ⚬ Providers still do not understand it ⚬ *“It’s starting to get mentioned but it doesn’t get the attention it deserves.”*
Concluding/Additional Remarks	• *“In an ideal world, there would be PCOS treatment clinics all around the world and all the providers would have the opportunity to converse about each patient.”* • *“PCOS is really calling for registered dietitians and can potentially increase the need for what we do and really help a lot of people.”* • *The better the clinician understands PCOS, the better they are able to treat it!*

## References

[1] Okoroh E.M., Hooper W.C., Atrash H.K., Yusuf H.R., Boulet S.L. (2012). Prevalence of polycystic ovary syndrome among the privately insured, united states, 2003–2008. Am. J. Obstet. Gynecol..

[2] March W.A., Moore V.M., Willson K.J., Phillips D.I., Norman R.J., Davies M.J. (2010). The prevalence of polycystic ovary syndrome in a community sample assessed under contrasting diagnostic criteria. Hum. Reprod..

[3] Futterweit W. (1999). Polycystic ovary syndrome: Clinical perspectives and management. Obstet. Gynecol. Surv..

[4] Azziz R., Carmina E., Dewailly D., Diamanti-Kandarakis E., Escobar-Morreale H.F., Futterweit W., Janssen O.E., Legro R.S., Norman R.J., Taylor A.E. (2009). The androgen excess and PCOS society criteria for the polycystic ovary syndrome: The complete task force report. Fertil. Steril..

[5] Broekmans F.J., Knauff E.A., Valkenburg O., Laven J.S., Eijkemans M.J., Fauser B.C. (2006). PCOS according to the rotterdam consensus criteria: Change in prevalence among WHO-II anovulation and association with metabolic factors. BJOG.

[6] Knochenhauer E.S., Key T.J., Kahsar-Miller M., Waggoner W., Boots L.R., Azziz R. (1998). Prevalence of the polycystic ovary syndrome in unselected black and white women of the southeastern united states: A prospective study. J. Clin. Endocrinol. Metab..

[7] Kauffman R.P., Baker V.M., Dimarino P., Gimpel T., Castracane V.D. (2002). Polycystic ovarian syndrome and insulin resistance in white and mexican american women: A comparison of two distinct populations. Am. J. Obstet. Gynecol..

[8] Moran L.J., Pasquali R., Teede H.J., Hoeger K.M., Norman R.J. (2009). Treatment of obesity in polycystic ovary syndrome: A position statement of the androgen excess and polycystic ovary syndrome society. Fertil. Steril..

[9] Moran L.J., Lombard C.B., Lim S., Noakes M., Teede H.J. (2010). Polycystic ovary syndrome and weight management. Women’s Health.

[10] Geier L.M., Bekx M.T., Connor E.L. (2012). Factors contributing to initial weight loss among adolescents with polycystic ovary syndrome. J. Pediatr. Adolesc. Gynecol..

[11] Lamb J., Closshey W., Huddleston H., Davis G., Zane L., Cedars M. (2007). A multidisciplinary polycystic ovarian syndrome (PCOS) clinic: A new model for care and research. Fertil. Steril..

[12] Teede H.J., Misso M.L., Deeks A.A., Moran L.J., Stuckey B.G., Wong J.L., Norman R.J., Costello M.F. (2011). Assessment and management of polycystic ovary syndrome: Summary of an evidence-based guideline. Med. J. Aust..

[13] Glaser B. (1978). Theoretical Sensitivity: Advances in the Methodology of Grounded Theory.

[14] Cartwright D., Zander A., Cartwright D., Zander A. (1968). The nature of group cohesivenss. Group Dynamics: Research and Theory.

[15] Terborg J., Castore C., DeNinno J. (1976). A longitudinal field investigaion of the impact of group composition on group performance and cohesion. J. Pers. Soc. Psychol..

[16] Haythorn W., Couch A., Haefner D., Langham P., Carter L. (1956). The behavior of authoritarian and equalitarian personalities in groups. Hum. Relat..

[17] Sapolsky A. (1960). Effect of interpersonal relationships upon verbal conditioning. J. Abnorm. Soc. Psychol..

[18] Braun V., Clarke V. (2006). Using thematic analysis in psychology. Qual. Res. Psychol..

[19] Moran L.J., Ko H.F., Misso M.F., Marsh K., Noakes M., Talbot M., Frearson M., Thondan M., Stepto N., Teede H.J. (2013). Dietary composition in the treatment of polycystic ovary syndrome: A systematic review to inform evidence-based guidelines. J. Acad. Nutr. Diet..

[20] Bonny A.E., Appelbaum H., Connor E.L., Cromer B., DiVasta A., Gomez-Lobo V., Harel Z.E., Huppert J., Sucato G., NASPAG Research Committee (2012). Clinical variability in approaches to polycystic ovary syndrome. J. Pediatr. Adolesc. Gynecol..

[21] Jeanes Y.M., Barr S.F., Smith K., Hart K.H. (2009). Dietary management of women with polycystic ovary syndrome in the United Kingdom: The role of dietitians. J. Hum. Nutr. Diet. Off. J. Br. Diet. Assoc..

[22] Humphreys L., Costarelli V. (2008). Implementation of dietary and general lifestyle advice among women with polycystic ovarian syndrome. J. R. Soc. Promot. Health.

[23] Bekx M.T., Connor E.C., Allen D.B. (2010). Characteristics of adolescents presenting to a multidisciplinary clinic for polycystic ovarian syndrome. J. Pediatr. Adolesc. Gynecol..

